# Association of *MnSOD* AA Genotype with the Progression of Prostate Cancer

**DOI:** 10.1371/journal.pone.0131325

**Published:** 2015-07-06

**Authors:** Taro Iguchi, Ching Y. Wang, Nicolas B. Delongchamps, Minoru Kato, Satoshi Tamada, Takeshi Yamasaki, Gustavo de la Roza, Tatsuya Nakatani, Gabriel P. Haas

**Affiliations:** 1 Department of Urology, SUNY Upstate Medical University, Syracuse, New York, United States of America; 2 Department of Urology, Osaka City University Graduate School of Medicine, Osaka, Japan; 3 Department of Urology, Cochin Hospital, Paris, France; 4 Department of Pathology, SUNY Upstate Medical University, Syracuse, New York, United States of America; Innsbruck Medical University, AUSTRIA

## Abstract

**Purpose:**

To investigate whether manganese superoxide dismutase (*MnSOD)* genetic polymorphism is associated with the clinical significance of prostate cancer.

**Materials and Methods:**

Prostates were obtained from 194 deceased men 45 years or older who did not have a history of prostate cancer. Serial sections and histological examinations of the prostate were performed. The *MnSOD* genotypes of the specimens were determined by polymerase chain reaction restriction fragment length polymorphism analysis.

**Results:**

Of the 194 men, 31 and 26 had clinically insignificant and significant prostate cancer. Clinically significant cancer comprised 29% and 58% of the cancers in men <70 and >70 years old, respectively. The age-specific proportion of significant cancer significantly increased with the advance of age (*p*<0.001). *MnSOD* AA, as compared with the other genotypes (VA and VV together), was associated with significant prostate cancer across all ages, odds ratio (OR) 2.34, 95% confidence interval (CI) 0.99-5.49, and in men older than 69 years (OR 4.89, 95% CI 1.51-15.8), but not in men younger than 70 years. The genotype was not associated with clinically insignificant cancer regardless of age. The comparison between significant and insignificant cancer, the OR (95% CI) for *MnSOD* AA was 5.04 (1.05-24.2) (sensitivity 0.57, specificity 0.78, positive predictive value 0.78) in men older than 69 years.

**Conclusions:**

*MnSOD* polymorphism is strongly associated with the clinical significance of prostate cancer in men older than 69 years, but not in men younger than 70 years suggesting that oxidative stress may be involved in the progression of the disease. *MnSOD* may be a clinically useful marker to predict the potential of progression of prostate cancer.

## Introduction

Prostate cancer (PCa) is the most prevalent noncutaneous malignancy in men [1]. American men have an approximately 16% chance of being diagnosed with PCa in their lifetime with a lifetime risk of death at 2.6% and the incidence rate of PCa gradually increases from age 40 through the 70s, then gradually decreases in the older population according to Surveillance, Epidemiology, and End Results Program (SEER, http://seer.cancer.gov/statfacts/html/prost.html) [2]. Men 60 or younger who have not been diagnosed with PCa have a greater than 15% chance of being diagnosed with this cancer over their remaining lifespan and this probability decreases to less than 10% in men older than 75 [3]. However, the prevalence of PCa observed in autopsy cases is related to age, reaching 60% in men older than 80 [3]. The prevalence of insignificant cancer is greater than that of significant cancer in younger men, but this trend is reversed in older men [4].

Manganese superoxide dismutase (MnSOD) is present in the mitochondria and plays an important role in mitigating reactive oxygen species–mediated DNA damage by converting superoxide radicals to oxygen and hydrogen peroxide [5]. The human *MnSOD* gene has a polymorphism at codon 16, which encodes either alanine (A) or valine (V) [6]. *MnSOD* AA has been reported to be associated with a risk of breast cancer [7], esophageal cancer [8], and cervical cancer [9]. Some studies showed that *MnSOD* AA is associated with a decreased risk of liver carcinoma [10], lung cancer [11], and bladder cancer [12]. Recent meta-analysis showed that *MnSOD* AA contributed to a significantly increased risk of breast cancer among premenopausal women with lower antioxidant consumption [13].

The *MnSOD* AA genotype has been associated with PCa in smokers [14, 15] and men with low antioxidant status [16–18] or high iron intake [19]. However, three recent meta-analyses have offered conflicting results regarding the association between *MnSOD* gene polymorphism and the risk of PCa [13, 20, 21]. The latest meta-analysis indicated a low-penetrance susceptible gene in PCa development [20].

Conventional epidemiological case-control studies on PCa have not considered that up to 40% of control group patients may have undetected insignificant or significant PCa [3] and that even control subjects who are currently free from PCa may develop PCa in the future. We have reported that the contamination of control populations by undetected PCa reduces the reliability of study results and that rigorous characterization of the control group is needed to preserve the integrity of any conclusions [22]. As most insignificant PCa cases are not included in such analyses, these studies mainly investigate factors that may lead to the progression of insignificant PCa into significant PCa. We have previously reported the association of *MnSOD* AA and PCa in autopsy cases in which PCa status was ascertained by histological examination of serial sections of the prostate [23]. The present study investigated whether *MnSOD* polymorphism is associated with the significance of PCa.

## Materials and Methods

The use of samples from deceased individuals was exempted from the Institutional Review Board of SUNY Upstate Medical University. The specific samples used in this study have been described in previous publication [23]. We collected 194 prostates from consecutive autopsies of deceased men aged 45 and older with no known history of PCa from the University Hospital, Syracuse, NY, the Onondaga County Medical Examiner, Syracuse, NY, and the National Disease Research Interchange, Philadelphia, PA. Of these men, 178 were white, 7 African American, 1 Hispanic, and 8 of unknown origin.

The entire prostate was fixed and serially sectioned at 5-mm intervals. Each section was then embedded in paraffin, cut into 5-μm sections, and stained with hematoxylin-eosin. The tumor volume was calculated by multiplying each tumor surface by the section thickness and then multiplying this total by 1.5 to compensate for tissue shrinkage, as previously described [4]. The diagnosis of PCa was made by a single pathologist based on the same criteria used to diagnose clinical cases. All cancers detected were Gleason score of 3 or more. Tumors were defined as insignificant if they were organ-confined, had a volume of less than 0.5 cm^3^, and received a Gleason score of 6 or less [24, 25]. Conversely, tumors that were 0.5 cm^3^ or larger, Gleason’s score more than 6 or non-organ confined were defined as meeting histological criteria of clinically significant disease. [24, 25]. In the case of multifocal tumors, clinical significance was assigned if either single tumor exceeded 0.5 cm^3^.

DNA was extracted from the autopsied prostate tissues using QIAamp DNA Blood Mini Kits (Qiagen, Valencia, CA, USA) according to the supplier's instructions. The *MnSOD* gene was amplified by the polymerase chain reaction (PCR) and genotyped by PCR restriction fragment length polymorphism analysis, as described previously [23].

An unconditional logistic regression model was used for statistical analysis. Wald’s 95% confidence intervals (95% CI) were determined. The software package JMP version 3.2.1, SAS Institute, Inc. (Cary, NC, USA) was employed for all analyses.

## Results and Discussion

**Fig 1:**
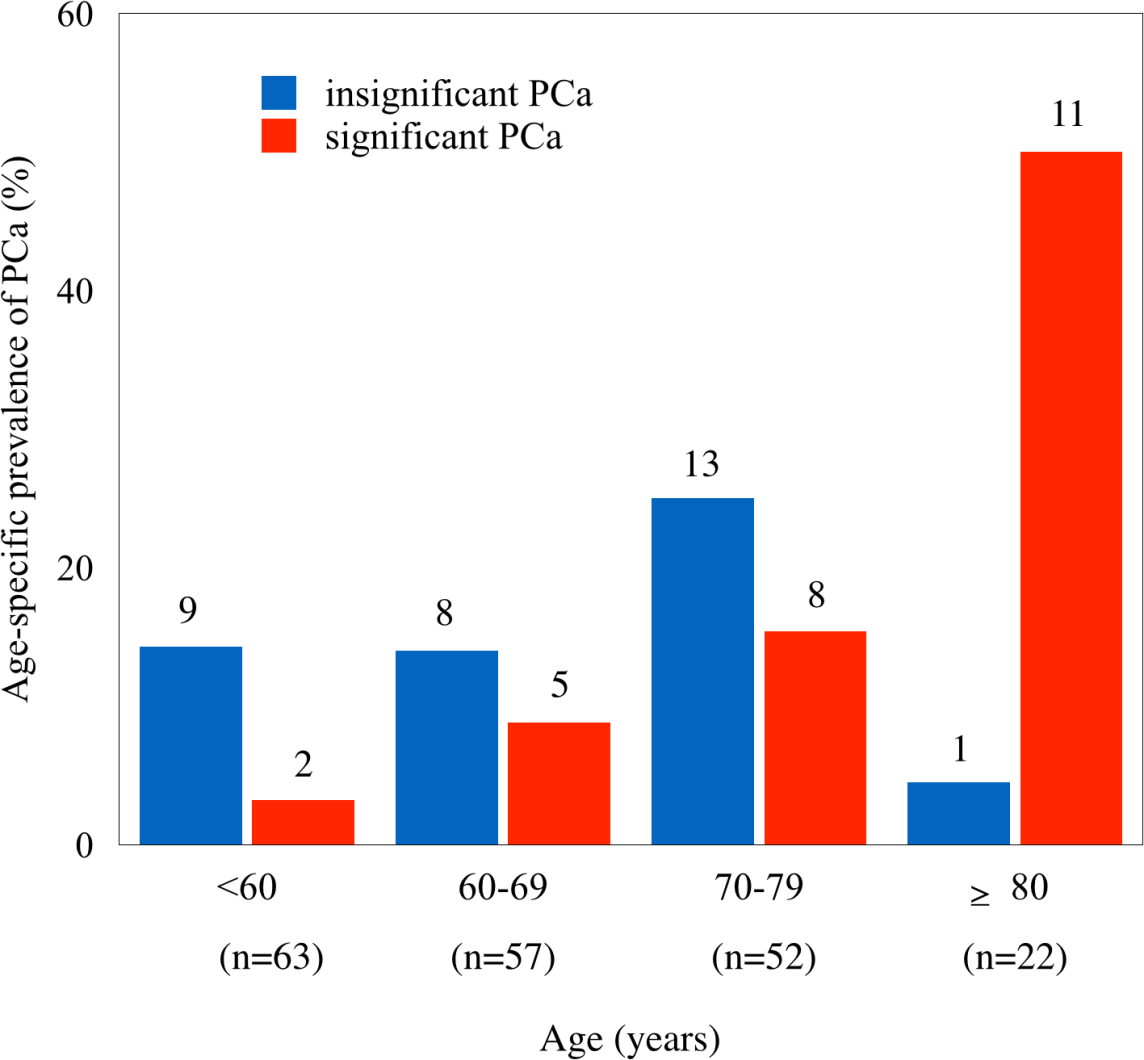
Age-specific prevalence of significant (red bar) and insignificant (blue bar) PCa. n = the number of total subjects in each group. The number on top of each bar is for the number of cases in each age group. The age-specific proportion of specific cancer (no. of significant cancers/no. of both significant and insignificant cancers) was significantly increased with the advance of age as analyzed by logistic regression (*p*<0.001).

Of the 194 men, 57 (29.4%) had PCa and 26 (13.4%) were clinically significant PCa. Among these clinically significant PCa, 20 had tumor size greater than 0.5 cm^3^, 20 had Gleason score >6, and only a single tumor demonstrated capsular invasion (S1 Table). Fig 1 shows the age-specific prevalence of significant and insignificant PCa. The prevalence of significant cancer increased with the advance of age. On the contrary the prevalence of insignificant cancer slightly increased with the advance of age but drastically decreased in men older than 79 years. The age-specific proportion of significant PCa significantly increased with the advance of age (*p*<0.001, Fig 1).

Because the median age at diagnosis of PCa is 67 years [2], we stratified our subjects into two groups, aged <70 years old and ≥70 years old. PCa was found in 24 of 120 (20.0%) and 33 of 74 (44.6%) men <70 and ≥70 years old, respectively. Clinically significant cancers represented 29.2% and 57.6% of the cancers in these age groups (Table 1).

**Table 1:** Association of *MnSOD* AA genotype and PCa in various age groups.

Age Group (years)		MnSOD genotype			
		AA	VA+VV	OR (95%CI)	OR (95%CI)
All	No PCa	41	96	1 (Ref.)	
	Insignificant PCa	11	20	1.29 (0.56–2.93)	1 (Ref.)
	Significant PCa	13	13	2.34 (0.99–5.49)	1.82 (0.62–5.27)
<70	No PCa	32	64	1 (Ref.)	
	Insignificant PCa	8	9	1.78 (0.62–5.04)	1 (Ref.)
	Significant PCa	2	5	0.80 (0.14–4.35)	0.45 (0.06–3.00)
≥70	No PCa	9	32	1 (Ref.)	
	Insignificant PCa	3	11	0.96 (0.22–4.24)	1 (Ref.)
	Significant PCa	11	8	4.89 (1.51–15.8)	5.04 (1.05–24.2)

We grouped the *MnSOD* VA and VV genotypes together, as both types do not appear to be a risk factor for PCa in comparison to AA [23]. As shown in Table 1, *MnSOD* AA was associated with significant PCa in men of all ages, especially in men older than 69 years. There was no such an association in men younger than 70 years. Neither an association was detected when comparisons were made for insignificant PCa with or without age stratification (Table 1).

In order to determine whether *MnSOD* AA can differentiate significant from insignificant cancer, we evaluated the use of this genotype for the test. As shown in Table 2, the test of this genotype had a reasonable specificity and positive predictive value for the group of men older than 69 years. However, it could not differentiate significant from insignificant cancer in the younger age group.

**Table 2:** Sensitivity, specificity and predictive value for *MnSOD* AA genotype for the differentiation of significant from insignificant PCa.

Age Group (years)	Sensitivity (95%CI)	Specificity (95%CI)	Positive Predictive Value (95%CI)	Negative Predictive Value (95%CI)	Positive likelihood ratio	Negative likelihood ratio
All	0.50 (0.29–0.70)	0.64 (0.45–0.81)	0.54 (0.32–0.74)	0.61(0.42–0.77)	1.41	0.78
<70	0.29 (0.04–0.71)	0.53 (0.27–0.77)	0.20 (0.025–0.56)	0.64 (0.35–0.87)	0.60	1.35
>70	0.57 (0.33–0.79)	0.78 (0.49–0.95)	0.78 (0.49–0.95)	0.57 (0.33–0.79)	2.70	0.53

Carcinogenesis is generally accepted to be a multiple-stage process. As seen in Fig 1, the ratio of significant PCa to insignificant PCa increased with age; it is therefore reasonable to assume that insignificant PCa does progress to significant PCa. Only one case of insignificant PCa was observed in a total of 12 PCa cases in men ≥80 years old, suggesting that most insignificant PCa cases eventually progress to significant PCa given sufficient time. Additionally, the observation that *MnSOD* AA was associated with significant PCa in older men (Table 1) suggests that MnSOD activity may be involved in the progression of prostate cancer. The observation that *MnSOD* AA was not associated with insignificant PCa suggests that oxidative stress may not be involved in the initiation of PCa. The lower clinical incidence of PCa in elderly men than in younger men may not be the result of a resistance to PCa, as previously suggested [24], but instead to underscreening in this population. Of 22 men older than 79 years in the present study, 10 did not have PCa. These men may not have been exposed to carcinogens or may have been genetically resistant to this cancer.

Previous epidemiological findings that the *MnSOD* AA genotype is associated with PCa in men with low antioxidant status [16–18] or high iron intake [19] indicate that oxidative stress could be a target for chemoprevention. More insignificant cancers than significant cancers are present in men, constituting 70% of undetected PCa in men 71 to 80 years old (Fig 1). Thus, preventive measures may be effective even for men in their 70s.

Mitochondria are the major source for ROS generation due to the continuous electron leakage at the mitochondrial transport chain. MnSOD is present in the mitochondria and plays an important role in mitigating reactive oxygen species–mediated DNA damage by converting superoxide radicals to oxygen and hydrogen peroxide [5]. Because Sutton et al showed that the A-containing MnSOD was transported more efficiently through the mitochondrial membrane [10], those who are AA genotype may have higher MnSOD activity as compared to those who are the other genotypes. In mitochondria, superoxide radical is converted by MnSOD into oxygen and hydrogen peroxide, which is further detoxified into water by glutathione peroxidase. The rate of hydrogen peroxide decomposition is proportional to both the glutathione level and the activity of glutathione peroxidase. The increased ability of superoxide radical detoxified due to the A allele may be beneficial with proper level of antioxidant. However, at a high concentration of peroxide, the step of NADP reduction becomes rate-limiting, and the overall reaction rate of the detoxification of peroxide is decreased [26]. Thus, high activity of MnSOD may lead to metabolic imbalance and induce toxicity if the rate of hydrogen peroxide decomposition is decreased. This might result in reducing ability to scavenge free radicals in mitochondria which would have the potential is to increase the risk for carcinogenesis. MnSOD may be particularly important in the prostate since the gland is thought to be rich in mitochondria[27]. Recently it is reported that MnSOD is upregulated in prostate during cancer progression and an inverse relation between MnSOD and androgen receptor, supporting the role of the mitochondrial enzyme in the acquirement of androgen-independence status [28].

With the increasing use of PSA in PCa screening, more insignificant PCa cases are being diagnosed. For example, up to 80% of PCa cases detected in the placebo group of the Prostate Cancer Prevention Trial had a Gleason score of ≤6 [29]. Nevertheless, the determination of whom should be treated remains an important issue. Although the *MnSOD* polymorphism had a low sensitivity (0.57) in the present study, it showed a reasonable specificity and positive predictive value (0.78) in predicting the progression of cancer to a case requiring immediate medical attention in men age 70 or older (Table 2).

Technically, all of the subjects in autopsy studies may be considered to have “clinically insignificant cancers” because these cancers were not diagnosed during the lifetime of the subjects, who deceased from unrelated causes. Had they been properly screened, judged on the histological evidence many of them should have been diagnosed prior to the death. The present work addresses several issues of importance regarding prostate carcinogenesis. Our findings support the hypothesis that oxidative stress may promote the development of small, well-differentiated cancers into large, more undifferentiated cancers. The U.S. Preventive Services Task Force (USPSTF) recommended against prostate-specific antigen (PSA) screening for PCa in men aged 75 years or older in 2008 [30]_,_ while the USPSTF’s new draft recommendations regarding PSA screening, issued in 2011, recommend against the use of the test in men under age 75 [31]. Although these recommendation are controversial, MnSOD germline testing could conceivably identify a subgroup of men who are at higher risk of aggressive PCa and, who could have a better risk:benefit ratio with PSA screening. *MnSOD* polymorphism may be an important biomarker to predict the progression of prostate cancers. This may be useful for detecting significant PCa in elderly men and may improve the results of screening when combined with PSA testing.

## Conclusions

*MnSOD* polymorphism is strongly associated with the clinical significance of prostate cancer in men older than 69 years, but not in men younger than 70, suggesting that oxidative stress may be involved in the progression of the disease. *MnSOD* AA genotype may have a clinical value to predict tumor progression of prostate cancer.

## Supporting Information

S1 Table Whole data of the study. Race: 0 for Caucasian; 1 for African American; 2 for others. PCa: 0 for no cancer; 1 for insignificant cancer; 2 for significant cancer. ITV: index tumor volume (mm^3^)
(XLSX)
